# Supplementary material for: Chemical Interaction among Termite-Associated Microbes
Source: J Chem Ecol. 2017 Nov 13;43(11):1078–85. doi: 10.1007/s10886-017-0900-6 (PMC5735195; doi:10.1007/s10886-017-0900-6)
Supplement: Supplementary file 1 — (DOCX 406601 kb) [file 10886_2017_900_MOESM1_ESM.docx]

CHEMICAL INTERACTION AMONG TERMITE-ASSOCIATED MICROBES

EMILY MEVERS^1^, THOMAS CHOUVENC^2^, NAN-YAO SU^2^, JON CLARDY^1^*

*^1^Department of Biological Chemistry and Molecular Pharmacology, Harvard Medical School, Boston, MA, 02115; ^2^Department of Entomology and Nematology, University of Florida, Ft. Lauderdale, FL, 33314.*

*To whom correspondence should be addressed. Tel: (617) 432-2845. Fax: (617) 432-3780. E-mail: [jon_clardy@hms.harvard.edu](mailto:jon_clardy@hms.harvard.edu)

**Abstract –** Bacteria and fungi in shared environments compete with one another for common substrates, and this competition typically involves microbially-produced small molecules. An investigation of one shared environmental niche, the carton material of the Formosan subterranean termite *Coptotermes formosanus*, identified the participants on one of these molecular exchanges. Molecular characterization of several termite-associated actinobacteria strains identified eleven known antimicrobial metabolites that may aid in protecting the *C. formosanus* colony from pathogenic fungal infections. One particular actinobacterial-derived small molecule, bafilomycin C1, elicited a strong chemical response from *Trichoderma harzianum*, a common soil saprophyte. Upon purification and structure elucidation, three major fungal metabolites were identified, t22-azaphilone, cryptenol, and homodimericin A. Both t22-azaphilone and homodimericin A are strongly upregulated, 123- and 38-fold, respectively, when exposed to bafilomycin C1, suggesting each play a role in defending *T.* *harzianum* from the toxic effect of bafilomycin C1.

**Key Words –** *Trichoderma harzianum*, *Coptotermes formasanus*, Termite, Actinobacteria, Induction.

INTRODUCTION

Throughout their evolutionary history insects have engaged in mutually beneficial associations with microbes, including bacteria belonging to the chemically rich class of actinobacteria (Kaltenpoth 2009; Caldera et al. 2009; Crawford and Clardy 2011; Nett et al. 2009). In paradigmatic insect-bacteria systems the insect provides essential nutrients to the microbe and the bacteria provides a fitness advantage to the insect, such as an efficient chemical defense. Previous reports have illustrated the ability of actinobacteria associated with a variety of insects – beetles, ants, termites, and beewolf wasps – to protect the insect hosts from infections by ecologically relevant pathogens (Scott et al. 2008; Oh et al. 2009a, b; Carr et al. 2012; Kaltenpoth et al. 2005; Kroiss et al. 2010). These studies exemplify the extraordinary potential of actinobacteria to produce structurally diverse antimicrobial agents, as illustrated by dentigerumycin, mycangimycin, and microtermolides (Oh et al. 2009a, b; Carr et al. 2012). In contrast, studies on the molecular basis of the microbial pathogens’ response to the actinobacterial defenses in an ecologically relevant framework are sparse.

The Formosan subterranean termite, *Coptotermes formosanus*, is an invasive and highly destructive insect, causing an estimated billion dollars per year in infrastructure damage and control cost in the United States alone (Lax and Osbrink 2003; Rust and Su 2012). This termite species builds large underground nests that can contain more than one million individuals with foraging tunnels up to 100 m (King and Spink 1969; Su and Scheffrahn 1988). Due to the sheer size of these nests, they can be difficult to control via conventional soil insecticide treatments (Su and Scheffrahn 1998) and biological control attempts failed to eliminate field colonies (Culliney and Grace 2000; Grace 1997; Lax and Osbrink 2003; Chouvenc et al. 2011). The core of the termite nest is filled with ‘carton material’, a sponge-like structure composed of nutritious chewed wood particles and fecal material that promotes the growth of mutualistic and opportunistic microbes, including parasites and pathogens (Chouvenc et al. 2013a). Preventing pathogenic fungi or other microbes from proliferating in the termite colony and on the fecal nest structure requires multiple mechanisms, including mutual- and self-grooming behavior by the termites to avoid bringing spores or fungal mycelia into the nest (Yanagawa et al. 2008; Chouvenc and Su 2010; Chouvenc and Su 2012). It also involves mutualistic relationships with beneficial microbes, especially the actinobacteria found within the carton material of the nests that significantly reduce the rate of infections by the fungal entomopathogen *Metarhizium anisopliae* (Chouvenc et al. 2013b).

We now report our recent efforts to evaluate the extracts from nine actinobacteria strains isolated from *C. formosanus* nests, that were previously identified to possess antifungal activity along with an initial examination of the fungal responses they induce (Chouvenc et al. 2013b). The current study led to the identification of eleven known biologically active bacteria-derived metabolites with antimicrobial activity. Interestingly, one *Streptomyces* metabolite induces the production of cryptenol (**1**), t22-azaphilone (**2**), and homodimericin A (**3**) in *Trichoderma harzianum* (Fig. 1), an ecologically relevant soil fungus that is known to take over the carton material of collapsed termite nests (Chouvenc et al. 2013a). The induction of these metabolites led to insights into the chemical interactions that are possibly occurring among microbes within the termite nest.

METHODS AND MATERIALS

*General Experimental Procedures.* Optical rotations were measured on a JASCO P-2000 polarimeter, circular dichroism spectrum on a JASCO J-815, and UV spectra on an Amersham Biosciences Ultrospec 5300-pro UV/Visible spectrophotometer. NMR spectra were recorded with benzene or dimethylsufoxide as internal standards (δ_C_ 128.06, δ_H_ 7.16 for C_6_H_6_ and δ_C_ 39.52, δ_H_ 2.50 for DMSO) on a Varian Oxford 600 MHz spectrometer equipped with a 5 mm AutoX HCN triple res inverse probe (600 and 150 MHz for ^1^H and ^13^C NMR, respectively), and on a Varian VNMRS (Varian NMR System) 500 MHz spectrometer equipped with a broadband Cold Probe (500 and 125 MHz for ^1^H and ^13^C NMR, respectively). LR-LCMS data were obtained using an Agilent 1200 series HPLC system equipped with a photo-diode array detector and a 6130 quadrupole mass spectrometer. HRESIMS was carried out using a Bruker Maxis Impact HD LC-q-TOF Mass Spectrometer equipped with an uHPLC system. HPLC purifications were carried out using Agilent 1100 or 1200 series HPLC systems (Agilent Technologies) equipped with a photo-diode array detector. All solvents were of HPLC quality.

*Actinobacteria Isolation and Culture Conditions.* Actinobacterial strains were isolated from the carton material of three colonies of the Formosan subterranean termite, *Coptotermes formosanus*, and the isolation protocol and characterization of these strains were reported previously (Chouvenc et al. 2013b). Nine of these actinobacterial strains – 7 *Streptomyces* and 2 *Kitasatospora* strains (Table 1) – were chosen for chemical investigations based on their interesting antimicrobial activity. *Streptomyces* sp. 4231 and *T. harzianum* WC13 (Genbank: KX694115) were both isolated from *C. formosanus* colonies, though were not found in the same colonies. *T. harzianum* WC13 was isolated from a dying *C. formosanus* colony and as the microbial community from the carton nest also collapsed, *T. harzianum* became the dominant saprophyte in a case of ecological succession (Chouvenc et al. 2013a). The actinobacterial strains were grown on 1/5 potato dextrose agar (DB Difco PDA) or 1/5 potato dextrose broth (DB Difco PDB) for 14 days. Solid agar cultures were kept at 30 ^o^C in an incubator, while liquid cultures were grown in shakers maintained at 30 ^o^C and 200 rpm.

*General Extraction and Isolation Procedure.* The solid agar plates for the actinobacteria and *T. harzianum* cultures were exhaustively extracted (5 – one hour soaks) using 2:1 dichloromethane (DCM)—methanol (MeOH), to afford a crude extract. Liquid cultures were centrifuged and the spent media was passed through a column of XAD-16 resin. The resin was subsequently rinsed with deionized water, and washed with MeOH to elute the organic material. The MeOH wash was dried using rotary evaporator, yielding a crude extract. All crude extracts were fractionated by reverse-phase (RP) solid phase extraction (SPE) using a stepwise gradient solvent system of decreasing polarity, starting from 75% water (H_2_O)/acetonitrile (ACN) to 100% DCM (five fractions, A-75% H_2_O/ACN, B-50% H_2_O/ACN, C-25% H_2_O/ACN, D-100% ACN, and E-100% DCM). All SPE fractions were tested for antimicrobial activity against *Bacillus subtillis*, a gram-positive bacterium and five soil fungi known to opportunistically be associated with termite nests (Zoberi and Grace 1990; Chouvenc et al. 2013a), *T. harzianum*, *Beauveria bassiana*, *Penicillum* sp., *Aspergillus nomius*, and *Metarhizium anisopliae*. SPE fractions that exhibited activity against any of the microbes were subsequently purified on RP HPLC using one of the following Phenomenex semi-preparative columns, Kinetex Biphyenyl, Luna C18(2), Phenyl-Hexyl, or Hydro-RP, resulting in the purification of 11 known secondary metabolites (Table 1 and Figs S18-S29).

*Purification of Streptomyces sp. 4231 Metabolites (Bafilomycins).* The D SPE fraction (100% ACN) from *Streptomyces* sp. 4231 grown on solid agar (1/5 PDA) induced pigment formation in *T. harzianum* and exhibited activity against *B. subtillis* and *A. nomius*, and consequently was subjected to for further purification by RP HPLC. Further fractionation using a Phenomenex 4 µm Luna C18(2) semi-preparative column, with a gradient from 50% ACN + 0.1% formic acid (FA)/H_2_O + 0.1% FA to 100% ACN + 0.1% FA over 25 min, yielded pure nataxazole, bafilomycin C1 (34.5 mg/L), B1, and D. The isolated material was confirmed to be bafilomycin C1 (**4**) via HRLCMS co-injections with authentic bafilomycin C1 (Enzo Scientific) (Fig S25).

*Preparation of Conditioned Media (CM) for Trichoderma harzianum WC13 and Isolation of Induced Metabolites.* The solid CM used in the induction studies was prepared following the previously reported method (Mevers et al. 2016). Briefly, the supernatant from liquid cultures of *Streptomyces* sp. 4231 was combined with fresh media (2/5 PDA, 3% agar) and subsequently used to make CM plates (55 mL; 150 x 15 mm Petri dish). These plates were then inoculated with refrigerated glycerol spore stocks (in 25% glycerol) from *T.* *harzianum* WC13 (Chouvenc et al. 2013a) and incubated at 30 ^o^C for 4 days. At this time the plates were extracted and preliminarily fractionated following the method described above. The B SPE fraction (50% ACN/H_2_O) appeared to be enriched with induced yellow pigments and was thus subjected to further purification by RP HPLC. The pigments were purified using a Phenomenex Kinetex 5 µm Biphenyl 100 Å semi-preparative column, with an isocratic gradient of 35% ACN + 0.1% FA/H_2_O + 0.1% FA, yielding 4.5 mg/L of culture media of cryptenol (**1**), 5.8 mg/L of culture media of t22-azaphilone (**2**), and 4.3 mg/L of culture media of homodimericin A (**3**). Induction levels for compounds **1**, **2**, and **3** were quantified from SPE fractions using extracted ion chromatograms (EIC) from LR-LCMS and determined to be 1.2-, 123-, and 38.7-fold increase in production, respectively, when grown in the presence of 30 μM bafilomycin C1 opposed to on 1/5 PDA for the same amount of time. The bafilomycin C1 containing solid agar plates were prepared by the addition of 1 mL of a 3 mM solution of bafilomycin C1 in DMSO to 1 L of freshly autoclaved 1/5 PDA media.

*Determining Relative Configuration of Cryptenol* *Using Acetonide Protection.* An aliquot of cryptenol (**1**, 0.25 mg) was dissolved in 300 µL of ethanol (EtOH) and treated with a small amount of 10% Pd/C and H_2_ (g) at room temperature (rt) for 4 h. at which point the reaction mixture was filtered through a glass plug containing celite. The reaction product was dried under vaccum, then dissolved in 0.6 mL (3 x 0.2 mL rinses) of dry DCM and transfered to a 10 mL pear flask that was previously charged with a small amount of activated 3Å molecular sieves. Subsquently, the pear flask was treated with *p*-toluenesulfonic acid monohydrate (28 µg, 0.15 µmol) in 0.2 mL of DCM and 2,2-dimethoxypropane (4 µL). The reaction mixture was stirred at room temperature for 16 h. At which point, 1 mL of saturated NaHCO_3_ (aq) was added and the mixture was extracted three times with DCM. The organic layer was dried using Na_2_SO_4_, filtered over celite, and dried under vacuum. The reaction product was then analyzed by ^1^H NMR using a 3 mm NMR tube (160 µL) in C_6_D_6_. The ^1^H NMR spectrum of the product was compared (Fig. S8) to two standards – threo-5,6-dodecanediol and erythro-5,6-dodecanediol – which were synthesized following the above procedure, assigning the relative stereoconfiguration as erythro.

**Cryptenol (1):** pale yellow oil; [α]^26^_D_ -16.8 (*c* 0.34, MeOH); UV (MeOH) *λ*_max_ (log ε) 232 (4.11), 255 (4.20), 265 (4.27), 275 (4.20) nm; NMR (600 MHz, CDCl_3_) and ^13^C NMR (125 MHz, CDCl_3_) see table 2; HRESIMS [M+Na]^+^ *m/z* 229.1196 (calcd for C_13_H_18_NaO_2_ 229.1205, Δ 3.9 ppm). See supporting inforamtion figures S1-S8.

**T22-azaphilone (2):** amorphous yellow solid; [α]^26^_D_ -70.6 (*c* 0.31, MeOH); UV (MeOH) *λ*_max_ (log ε) 208 (2.87), 282 (2.80), 355 (2.95) nm; NMR (600 MHz, C_6_D_6_) 7.21 (1H, s), 6.53 (1H, dd; 15.2, 11.0), 5.86 (1H, dd; 15.0, 11.0), 5.64 (1H, m), 5.50 (1H, s), 5.28 (1H, d; 15.2), 5.16 (1H, s), 4.24 (1H, m), 2.49 (1H, dd; 13.8, 9.0), 2.30 (1H, dd; 13.7, 3.2), 1.53 (3H, d; 6.8), 1.53 (3H, s), 1.08 (3H, d; 6.2); ^13^C NMR (125 MHz, C_6_D_6_) 192.0 x 2, 171.4, 155.4, 152.5, 141.5, 136.4, 135.3, 130.5, 119.5, 114.2, 109.5, 107.7, 85.1, 65.7, 43.0, 22.3, 21.8, 17.7; HRESIMS [M+H]^+^ *m/z* 345.1315 (calcd for C_19_H_21_O_6_ 345.1338, Δ 3.7 ppm). See supporting information figures S9-S16.

*Spot-on-lawn Antimicrobial and Induction Assays.* Antimicrobial potential of reduced complex fractions and pure compounds were assessed using a modified disc-diffusion assay. For fungal assays, an aliquot (75-120 μL) of the fungal spore stock (in 25% glycerol) was added to a 12 mL conical vial containing 10 mL of 1/5 PDA soft agar (55 ^o^C, 0.75% agar). The mixture was vortexed and then poured over a plate of 1/5 PDA (25 mL; 100 x 15 mm Petri dish). For the *B. subtilus* assays, 100 μL of an overnight culture (in LB) was added to 10 mL of the soft agar, and was similarly vortexed and poured over a LB plate. Upon solidifying (~10 min), 1.5 μL of each fraction or pure compound resuspended in 50% ACN/H_2_O at 10 mg/mL, was spotted on the agar along with a solvent control, trying to minimize the surface diffusion. The inoculated assay plates were subsquently incubated at 30 ^o^C for either 24 h or 72 h for the bacteria and fungi, respectively, at which point the zones of inhibition were measured and photgraphed. Determining the induction potential of fractions from *Streptomyces* sp. 4231 were assessed in a similar manner. See previous publication for detailed method (Mevers et al. 2016).

*Determining Minimum Inhibition Concentration of T22-azaphilone on Solid Agar.* A parent stock solution of freshly purified t22-azaphilone was prepared by dissolving an aliquot in DMSO at 15 mg/mL. A subsquent 1:10 dilution with 100% water yielded a 1.5 mg/mL solution in 10% DMSO (aq). This solution was then further diluted to form six working stock solutions with the final concentrations of 1.5, 0.75, 0.375, 0.190, 0.094, and 0.047 mg/mL. A 0.5 mL aliquot of each of these working solutions was added to a 50 mL conical vial containing 14.5 mL of 1/5 PDA (55 ^o^C; 1.5% agar). The solutions were vortexed and dispensed in 1 mL aliquots per well into a 24-well sterile plate with each plate containing three wells per concentration. In addition to the six concentrations of t22-azpahilone, three controls (two wells each) were plated on every plate – DMSO control (0.5 mL of 10% aqueous DMSO), negative control (1/5 PDA), and positive control (Penicillin-Streptomycin at 500 units and 0.5 mg/mL, respectively). Upon solidifiying, each well was inoculated with 1 μL of one of the bacterial suspension, *Streptomyces* sp. 4231 and 2083B and *Kitasatospora* sp. 2404, adjusted to 1 x 10^8^ cfu/mL (Wiegand et al. 2008). Plates were incubated for 48 h at 30 ^o^C, at which point the plates were analyzed for bacterial growth and photographed.

RESULTS

*Identification of Antimicrobial Agents Produced by Termite-Associated Actinobacteria –* Nine termite-associated actinobacterial strains – 7 *Streptomyces* and 2 *Kitasatospora* strains (Table 1) – were chosen for chemical investigations based on their antimicrobial activity, previously reported in Chouvenc et al (2013b). These strains exhibited a range of inhibitory activity against both bacteria and ecologically relevant soil fungi in a binary competition assay. Upon extraction of either solid or liquid cultures from each strain and successive rounds of reverse-phase (RP) chromatography, eleven known metabolites were identified, including fungichromin, dactinomycin, 16-deethylindanomycin, streptonigrin, berninamycin A and B, bafilomycin B1, C and D, nataxazole, and mycotrienin (Table 1 and Figs S18-S29). Several new analogs of dactinomycin, berninamycin, and 16-deethylindanomycin were observed by mass spectrometry but were not isolated or characterized. Of this diverse group, only one of the metabolites (fungichromin) exhibited selective antifungal activity, four compounds (dactinomycin, berninamycin A and B, and streptonigrin) exhibited antibacterial activity against *B. subtilus*, and five metabolites (bafilomycin B1, C1 and D, mycotrienin, and 16-deethylindanomycin) exhibited both antibacterial and antifungal activity in spot-on-lawn assays, which agreed with previous literature on each of the secondary metabolites (Hollstein 1974; Shih et al. 2003; Reusser 1969; Rao et al. 1963; Sugita et al. 1982; Yueda et al. 1995; Bowman et al. 1988; Larsen et al. 1988).

*Induction and Identification of Antibacterial Metabolites Produced by T. harzianum WC13 –* One particularly interesting binary competition assay revealed a chemical interaction between *Streptomyces* sp. 4231 and *T. harzianum* WC13, where a small molecule produced and excreted by the bacteria changed the growth morphology and upregulated the production of pigments in the fungus (Fig. 2). Bioassay-guided isolation identified bafilomycin C1 (**4**), a well-known antifungal agent, as the bacterial-derived small molecule responsible for inducing pigment formation, but two structural analogs, bafilomycin B1 and D, exhibited no appreciable ability to elicit this response (Figs S31-S32).

Identification of the upregulated fungal metabolites required largescale cultivation of *T. harzianum* WC13 on solid media that was conditioned with either sterilized spent liquid media from a 14-day culture of *Streptomyces* sp. 4231 or with purified bafilomycin C1 (30 μM). In both cases, the growth of *T. harzianum* WC13 was significantly stunted, with almost no visible mycelia formation or sporulation, though robust pigmentation of the agar was observed after just four days. Extraction of the solid agar plates and subsequent rounds of RP HPLC purification yielded the isolation of three natural products, cryptenol (**1**), t22-azaphilone (**2**), and homodimericin A (**3**). Both t22-azaphilone (**2**) and homodimercin A (**3**) have been previously reported (Vinale et al. 2006; Vinale et al. 2009; Mevers et al. 2016). The production of both **2** and **3** are strongly upregulated, approximately 123- and 38-fold, respectively, when grown in the presence of 30 μM of bafilomycin C1, while the production of **1** remains relatively unchanged (1.2-fold increase) (Fig. S17). Subsquently, all three metabolites were found to be consitutively expressed in *T. harzianum* T22 (ATCC 20847), a strain that is commonly used as a biocontrol agent (Vinale et al. 2006).

Cryptenol (**1**) was isolated as a pale-yellow oil having a molecular formula C_13_H_18_O_2_ based on ESI-QTOF data and requiring five degrees of unsaturation (Fig. S7). The ^1^H and ^13^C NMR data suggested the pressence of five olefins and are responsible for all degrees of unsaturation with 10 carbon resonances between 117 and 138 ppm (δ_C_ 137.1, 136.1, 133.3, 132.1, 132.0, 131.4, 130.0, 129.6, 128.0, and 117.1), and 11 proton resonances between 5.0 and 6.4 ppm (Fig. S2). Planar structure elucidation was greatly facilitated by the fact that cryptenol contains only one spin-system that could easily be pieced together using gH2BC correlations. Further analysis of the complete 2D NMR dataset [gHSQC, gCOSY, gc2HMBC, and gH2BC (Table 2 and Fig. S1-S6)] led to the identification of the planar structure of cryptenol as trideca-2,4,8,10,12-pentaene-6,7-diol (Fig. 1).

The configuration of the four internal olefins of **1** were determined by ^3^*J* coupling constant analysis, while the relative configuration of the diol was determined using semi-synthetic methods. Measured coupling constants of 15.3 (H-2 and H-3), 15.3 (H-4 and H-5), 15.0 (H-8 and H-9), and 14.9 Hz (H-10 and H-11) are all indicative of *E* configurations for the four internal olefins. The relative configuration of the diol at C-6 and C-7 was determined by preparing the acetonide analog and comparing proton chemical shifts of H-6/H-7, as well as the introduced dimethyl moiety in the acetonide, to two synthetic standards. The acetonide analog of cryptenol was prepared by first reducing all of the olefins with 10% Pd/C and H_2_ (g), and then reacting with 2,2-dimethoxypropane. ^1^H NMR analsysis of the derivatived natural product led to the relative assingment of erythro (Fig. S8).

Cryptenol (**1**), homodimericin A (**3**), and t22-azaphilone (**2**) were each evaluated for antimicrobial activity against a range of ecologically relevant microbes, including eight strains of termite-associated actinobacteria, and six soil fungi (*T. harzianum*, *A. nomius*, *Penicillium* sp., *M. anisopliae*, *Trichoderma viride*, and *B. bassiana*). T22-azaphilone exhibited moderate antibacterial activity against every actinobacteria strain tested, including *Streptomyces* 4231, the bafilomycin producer with MICs ranging from 5-25 µg/mL (Fig. S34). No toxicity against any of the fungal pathogens was observed. Cryptenol and homodimericin A were found to be inactive against all microbes screened up to a maximum of 30 µg per spot in a spot-on-lawn assay (Fig. S33).

DISCUSSION

The carton material in a subterranean termite nest defines a specialized niche, as the fecal material provides nutrients for the growth of a diverse profile of microbes, including chemically-rich actinobacteria that provide chemical defenses against other microbes. Our earlier study showed that a termite colony greatly benefits from the presence of a wide variety of actinobacterial strains present in the carton in preventing infections from pathogenic soil fungi. In fact, inclusion of just one strain of biologically active actinobacteria to the carton material significantly reduces *M. anisopliae* infection rates to termite colonies (Chouvenc et al. 2013b). However, the microbes within the carton material are also in a constant competition with one another for limited nutrients and this continuing competition would be expected to drive a chemical interaction between the competing microbes. The molecules underlying this chemical interaction would have a range of functions from chemical defense to signaling to acquisition of nutrients. The strong selection pressure on termite-associated actinobacteria to develop an effective and diverse suite of biologically active small molecules is supported by previous studies (Carr et al. 2012; Kang et al. 2016).

Most of the research into insect-associated microbes has been focused on the toxic metabolites produced by the symbiotic bacteria, which have inhibitory activity against ecologically relevant pathogenic fungi with little attention being paid to the chemical response of the pathogen. Constant exposure to an antibiotic pressures the pathogen to evolve response mechanisms, such as detoxification of select antimicrobials, counters to the general effects of antimicrobial such as reactive oxygen species (ROS), repression of antibiotic biosynthesis in the producing organism, active efflux of the antibiotic, and/or production of antibiotics to neutralize the chemical defenses (Duffy et al. 2003; Brakhage and Schroeckh 2011). In this study, we propose that *T. harzianum* WC13 is capable of upregulating the production of its own small molecules in order to counter the effects of the bacterial derived antifungal agent. The release of bafilomycin C1 by *Streptomyces* sp. 4231 induces the production of several natural products – homodimericin A (**3**), t22-azaphilone (**2**), and cryptenol (**1**) by *T. harzianum* WC13. The prodigious upregulation of homodimericin A and t22-azaphilone production suggests that both have an ecological role in defending *T. harzianum* from the effects of bafilomycin C1. The bafilomycins are known potent and specific inhibitors of fungal vacuolar-type H^+^-ATPases, which when inhibited causes increases in both cellular pH and oxidative stress (Werner et al. 1983; Dröse and Altendorf 1997). We suggest that the production of homodimericin A, which requires a six-electron non-enzymatic oxidation from its starting material (Mevers et al. 2016; Ma et al. 2017), would relieve the oxidative stress caused by bafilomycin C1, while t22-azaphilone neutralizes the active threat by inhibiting the growth of *Streptomyces* sp. 4231 and thus ceasing the production of bafilomycin C1.

Our study reveals that the interactions among microbes within a subterranean termite nest are far more complex than previously thought. A subterranean termite colony represents an important resource of essential nutrients for a diverse array of microbes, including strains that are both beneficial and detrimental to the termites. While antibiotic-producing actinobacteria protect the colony from harmful pathogenic fungal infections, some fungi can develop mechanisms to overcome the antibiotic’s effect, such as how *T. harzianum* WC13 upregulates the production of homodimericin A and t22-azaphilone in order to combat and neutralize the effect of bafilomycin C. This observation may explain how *T. harzianum* WC13 is able persist in a termite’s nest and saprophytically take over the nest resource once the termite colony and associated actinobacteria community have collapsed (Chouvenc et al. 2013a). This study demonstrates that interactions among microbes in a termite nest is not bipartite, but a complex multipartite system where all organisms compete for resources in antagonistic and beneficial ways.

*Acknowledgment* – The work was funded by the National Institute of Health (RO1 GM086258 and U19 AI109673). We thank the HMS East Quad NMR Facility, the Harvard FAS Division of Science Core Facility, the ICCB-Longwood Screening Facility, and the Center for Macromolecular Interactions for assistance in acquiring analytical data.

REFERENCES

Bowman EJ, Siebers A, Altendorf K (1988) Bafilomycins: a class of inhibitors of membrane ATPases from microorganisms, animal cells, and plant cells. Proc Nat Acad Sci USA 85: 7972-7976. doi: 10.1073/pnas.85.21.7972

Brakhage AA, Schroeckh V (2011) Fungal secondary metabolites – Strategies to activate silent gene clusters. Fungal Genet Biol 48: 15-22. doi: 10.1016/j.fgb.2010.04.004

Caldera EJ, Poulsen M, Suen G, Currie CR (2009) Insect symbioses: A case study of past, present, and future fungus-growing ant research. Environ Entomol 38: 78-92. doi: 10.1603/022.038.0110

Carr G, Poulsen M, Klassen JL, Hou Y, Wyche TP, Bugni TS, Currie CR, Clardy J (2012) Microtermolides A and B from termite-associated *Streptomyces* sp. and structural revision of vinylamycin. J Org Lett 14: 2822-2825. doi: 10.1021/ol301043p

Chouvenc T, Su N-Y (2010) Apparent synergy among defense mechanisms in subterranean termites (Rhinotermitidae) against epizootic events: Limits and potential for biological control. J Econ Entomol 103: 1327-1337. doi.org/10.1603/EC09407

Chouvenc T, Su N-Y (2012) When subterranean termites challenge the rules of fungal epizootics. PLoS One 7: p.e34484. doi: 10.1371/journal.pone.0034484

Chouvenc T, Bardunias P, Efstathion CA, Chakrabarti S, Elliott ML, Giblin-Davis R, Su N-Y (2013a). Resource opportunities from the nest of dying subterranean termite (Isoptera: Rhinotermitidae) colonies: A laboratory case of ecological succession. Ann Entomol Soc Am 106: 771-778. doi: 10.1603/AN13104

Chouvenc T, Efstathion CA, Elliott ML, Su N-Y (2013b) Extended disease resistance emerging from the faecal nest of a subterranean termite. Proc R Soc B 280: 20131885. doi: 10.1098/rspb.2013.1885

Chouvenc T, Su N-Y, Grace K (2011) Fifty years of attempted biological control of termites – Analysis of the failure. Biol Control 59: 69-82. doi: 10.1016/j.biocontrol.2011.06.015

Culliney TW, Grace JK (2000) Prospects for the biological control of subterranean termites (Isoptera: Rhinotermitidae), with special reference to *Coptotermes formosanus*. B Entomol Res 90: 9-21. doi: 10.1017/S0007485300000663

Crawford JM, Clardy J (2011) Bacterial symbionts and natural products. Chem Commun 47:7559-7556. doi: 10.1039/c1cc11574j

Dröse S, Altendorf K (1997) Bafilomycins and concanamycins as inhibitors of V-ATPases and P-ATPases. J Exp Biol 200: 1-8. doi:

Duffy B, Schouten A, Raaijmakers JM (2003) Pathogen self-defense: Mechanisms to counteract microbial antagonism. Annu Rev Phytopathol 41: 501-538. doi: 10.1146/anurev.phyto.41.052002.095606

Grace JK (1997) Biological control strategies for suppression of termites. J Agric Entomol 14: 281-289.

Hollstein U (1974) Actinomycin. Chemistry and mechanism of action. Chem Rev 74: 625-652. doi: 10.1021/cr60292a002

Kaltenpoth M (2009) Actinobacteria as mutualists: general healthcare for insects? Trends Microbiol 17: 529-535. doi: 10.1016/j.tim.2009.09.006

Kaltenpoth M, Göttler W, Herzner G, Strohm E (2005) Symbiotic bacteria protect wasp larvae from fungal infestation. Curr Biol 15: 475-479. doi: 10.1016/j.cub.2004.12.084

Kang HR, Lee D, Benndorf R, Jung WH, Beemelmanns C, Kang KS, Kim KH (2016) Termisoflavones A-C, isoflavonoid glycosides from termite-associated *Streptomyces* sp. RB1. J Nat Prod 79: 3072-3078. doi: 10.1021/acs.jnatprod.6b00738

King EG, Spink WT (1969) Foraging galleries of the Formosan subterranean termite, *Coptotermes formosanus*, in Louisiana. Ann Entomol Soc Am 62: 536-542. doi: 10.1093/aesa/62.3.536

Kroiss J, Kaltenpoth M, Schneider B, Schwinger MG, Hertweck C, Maddula RK, Srohm E, Svatoš A (2010) Symbiotic streptomycetes provide antibiotic combination prophylaxis for wasp offspring. Nat Chem Biol 6: 261-263. doi: 10.1038NCHEMBIO.331

Larsen SH, Boeck LD, Mertz FP, Paschal JW, Occolowitz JL (1988) 16-Deethylindanomycin (A83094A), a novel pyrrole-ether antibiotic produced by a strain of *Streptomyces setonii*. Taxonomy, fermentation, isolation and characterization. J Antibiot 41: 1170-1177. doi: 10.7164/antibiotics.41.1170

Lax AL, Osbrink WL (2003) United States Department of Agriculture – Agriculture Research Service research on targeted management of the Formosan subterranean termite *Coptotermes formosanus* Shiraki. Pest Manag Sci 59: 788-800. doi: 10.1002/ps.721

Ma D, Liu Y, Wang Z (2017) Biomimetic total synthesis of (±)-homodimericin A. Angew Chem Int Ed 56: 7886-7889.

Mevers E, Saurí J, Liu Y, Moser A, Ramadhar TR, Varlan M, Williamson RT, Martin GE, Clardy J (2016) Homodimericin A: A complex hexacyclic fungal metabolite. J Am Chem Soc 138: 12324-12327. doi: 10.1021/jacs.6b07588

Nett M, Ikeda H, Moore BS (2009) Genomic basis for natural product biosynthetic diversity in the actinomycetes. Nat Prod Rep 26: 1362-1384. doi: 10.1039/b817069j

Oh DC, Poulsen M, Currie CR, Clardy J (2009a) Dentigerumycin: a bacterial mediator of an ant-fungus symbiosis. Nat Chem Biol 5: 391-393. doi: 10.1038/nchembio.159

Oh DC, Scott JJ, Currie CR, Clardy J (2009b) Mycangimycin, a polyene peroxide from a mutualist *Streptomyces* sp. Org Lett 11: 633-636. doi: 10.1021/ol802709x

Rao KV, Biemann K, Woodward RB (1963) The structure of streptonigrin. J Am Chem Soc 85: 2532-2533.

Reusser F (1969) Mode of action of berninamycin. An inhibitor of protein biosynthesis. Biochemistry-US 8: 3303-3308. doi: 10.1021/bi00836a026

Rusk MK, Su N-Y (2012) Managing social insects of urban importance. Annu Rev Entomol 57: 355-375. doi: 10.1146/annurev-ento-120710-100634

Scott JJ, Oh DC, Yuceer MC, Klepzig KD, Clardy J, Currie CR (2008) Bacterial protection of beetle-fungus mutualism. Science 322: 63. doi: 10.1126/science.1160423

Shih HD, Liu YC, Hsu FL, Mulabagal V, Dodda R, Huang JW (2003) Fungichromin: a substance from *Streptomyces padanus* with inhibitory effects on *Rhizoctonia solani*. J Agric Food Chem 51: 95-99. doi: 10.1021/jf025879b

Sommer PS, Almeida RC, Schneider K, Beil W, Süssmuth RD, Fiedler HP (2008) Nataxazole, a new

benzoxazole derivative with antitumor activity produced by *Streptomyces* sp. Tü 6176. Antibiot

(Tokyo) 61: 683-686.

Su, N-Y, Scheffrahn RH (1988) Foraging population and territory of the Formosan subterranean termite (Isoptera: Rhinotermitidae) in an urban environment. Sociobiology 14: 353-359.

Su, N-Y, Scheffrahn RH (1998) A review of subterranean termite control practices and prospects for integrated pest management programs. Integr Pest Manag Rev. 3:1-13. doi: 10.1023/A:1009684821954

Sugita M, Natori Y, Sueda N, Furihata K, Seto H, Otake N (1982) Studies on mycotrienin antibiotics, a novel class of ansamycins. III. The isolation, characterization and structures of mycotrienols I and II. J Antibiot 35: 1474-1479. doi: 10.7164/antibiotics.35.1474

Vinale F, Ghisalberti EL, Sivasithamparam K, Marra R, Ritieni A, Ferracane R, Woo S, Lorito M (2009) Factors affecting the production of *Trichoderma* *harzianum* secondary metabolites during the interaction with different plant pathogens. Lett Appl Microbiol 48: 705-711. doi: 10.1111/j.1472-765X.2009.02599x

Vinale F, Marra R, Scala F, Ghisalberti EL, Lorito M, Sivasithamparam K (2006) Major secondary metabolites produced by two commercial *Trichoderma* strains active against different phytopathogens. Lett Appl Microbiol 43: 143-148. doi: 10.1111/j.1472-765X.2006.01939.x

Werner G, Hagenmaier H, Drautz H, Baumgartner A, Zähner H (1984) Bafilomycins, a new group of macrolide antibiotics. J Antibiot 37: 110-117. doi: 10.7164/antibiotics.37.110

Wiegand I, Hipert K, Hancock REW (2008) Agar and broth dilution methods to determine the minimal inhibitory concentration (MIC) of antimicrobial substances. Nat Protoc 3: 163-175. doi: 10.1038/nprot.2007.521

Yanagawa Y, Yokohari F; Shimizu S (2008) Defense mechanism of the termite, *Coptotermes formosanus* Shiraki, to entomopathogenic fungi. J Invertebr Pathol 97: 165-170. doi: 10.1016/j.jip.2007.09.005

Zoberi M, Grace JK (1990) Fungi associated with the subterranean termite *Reticulitermes flavipes* in Ontario. Mycologia 82: 289-294. doi: 10.2307/3759899

**Supporting Information Available:** ^1^H NMR, ^13^C NMR, COSY, HSQC, HMBC, and H2BC spectra in *d*_6_-DMSO for cryptenol (**1**). ^1^H NMR, ^13^C NMR, COSY, HSQC, and HMBC spectra in C_6_D_6_ for t22-azaphilone (**2**). Antimicrobial assay results for cryptenol, t22-azaphilone, fungichromin, dactinomycin and analog, 16-deethylindanomycin, streptonigrin, berninamycins, bafilomycins, nataxazole, and mycotrienin. Binary time-course assay results between *Streptomyces* 4231 and *Trichoderma harzianum* WC13.

FIG. 1 Structures of induced metabolites (1-3) by the soil saprophyte *T. harzianum* WC13 and the inducing agent (4) produced by termite-associated *Streptomyces* sp. 4231.

FIG. 2 Induction of pigment formation in *T. harzianum* WC13 when co-cultured with *Streptomyces* sp. 4231


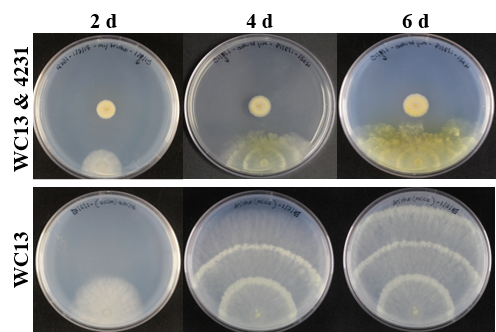


**Table 1.** Summary of natural products isolated from termite-associated actinobacteria and their reported antimicrobial activity

| **Active Metabolite** | **Producing Strain** | **Activity** | **Reference** |
| --- | --- | --- | --- |
| Fungichromin | 2392,^1^ 2545,^1^ 2338^1^ | Antifungal | Shih et al. 2003 |
| Dactinomycin | 2338, 2545 | Antibacterial | Hollstein 1973 |
| Bafilomycin C1 | 4231^1^ | Antifungal and Antibacterial | Bowman et al. 1988 |
| Bafilomycin B1 | 4231 | Antifungal and Antibacterial | Bowman et al. 1988 |
| Bafilomycin D | 4231 | Antifungal and Antibacterial | Bowman et al. 1988 |
| Nataxazole | 4231 | inactive | Sommer et al. 2008 |
| 16-deethylindanomycin | 4233^1^ | Antifungal and Antibacterial | Larsen et al. 1988 |
| Mycotrienin | 4326^1^ | Antifungal and Antibacterial | Sugita et al. 1982 |
| Berninamycin A | 2083B^1^ | Antibacterial | Reusser 1969 |
| Berninamycin B | 2083B | Antibacterial | Reusser 1969 |
| Streptonigrin | 2404^2^ | Antibacterial | Rao et al. 1963 |
| ^1^*Streptomyces* sp.; ^2^*Kitasatospora* sp. | |  |  |

**Table 2. ^1^H and ^13^C NMR assignments for cryptenol (1) in *d_6_*-DMSO**

| **position** | **δ_C_ ^b^** | **δ_H_ (*J* in Hz)^a^** | **H2BC^a^** | **HMBC^a^** | **COSY^a^** |
| --- | --- | --- | --- | --- | --- |
| 1 | 17.8 | 1.71 (d; *6.7*) | 2 | 2, 3 | 2, 3 |
| 2 | 128.0 | 5.63 (dq; *15.3, 6.7*) | 1, 3 | 1, 4 | 1, 3 |
| 3 | 131.4 | 6.03 (dd; *15.3, 10.7*) | 2, 4 | 1, 5 | 1, 2, 4 |
| 4 | 130.0 | 6.11 (dd; *15.3, 10.7*) | 5 | 1, 2, 6 | 3, 5, 6 |
| 5 | 132.0 | 5.60 (dd; *15.3, 5.6*) | 4, 6 | 3, 6 | 4, 6 |
| 6 | 74.5 | 3.86 (t; *5.6*) | 5, 7 | 4, 5, 7, 8 | 4, 5 |
| 7 | 74.5 | 3.89 (t; *5.6*) | 6, 8 | 5, 6, 8, 9 | 8, 9 |
| 8 | 136.1 | 5.81 (dd; *15.0, 5.6*) | 7, 9 | 7, 10 | 7, 9 |
| 9 | 129.6 | 6.20 (dd; *15.0, 10.4*) | 8, 10 | 7 | 7, 8, 10 |
| 10 | 133.3 | 6.27 (dd; *14.9, 10.4*) | 9, 11 | 11, 12 | 9/11 |
| 11 | 132.1 | 6.23 (m) | 10 | 10, 12 | 10, 12, 13b |
| 12 | 137.1 | 6.36 (dt; *16.9, 10.1*) | 13 | 10 | 11, 13a, 13b |
| 13a | 117.1 | 5.22 (d; *16.9*) | 12 | 11, 12 | 10, 12, 13b |
| 13b |  | 5.07 (d; *10.1*) | 12 | 11, 12 | 11, 12, 13a |
| OH(a) |  | 4.87 (bs) |  |  |  |
| OH(b) |  | 3.42 (bs) |  |  |  |

^a^ 600 MHz for ^1^H NMR, H2BC, HMBC, and COSY

^b^ 125 MHz for ^13^C NMR
